# The application of selective reaction monitoring confirms dysregulation of glycolysis in a preclinical model of schizophrenia

**DOI:** 10.1186/1756-0500-5-146

**Published:** 2012-03-15

**Authors:** Daniel Martins-de-Souza, Murtada Alsaif, Agnes Ernst, Laura W Harris, Nancy Aerts, Ilse Lenaerts, Pieter J Peeters, Bob Amess, Hassan Rahmoune, Sabine Bahn, Paul C Guest

**Affiliations:** 1 Dept of Chemical Engineering and Biotechnology, University of Cambridge, Tennis Court Road, Cambridge CB2 1QT, UK; 2 Janssen Research & Development, Division of Janssen Pharmaceutica N.V, Beerse, Belgium; 3 Dept of Neuroscience, Erasmus Medical Centre, Rotterdam, The Netherlands

**Keywords:** SRM, MRM, Multiplex, Schizophrenia, Proteomics, Preclinical, Assay, Glycolysis, Multiple reaction monitoring, Selective reaction monitoring

## Abstract

**Background:**

Establishing preclinical models is essential for novel drug discovery in schizophrenia. Most existing models are characterized by abnormalities in behavioral readouts, which are informative, but do not necessarily translate to the symptoms of the human disease. Therefore, there is a necessity of characterizing the preclinical models from a molecular point of view. Selective reaction monitoring (SRM) has already shown promise in preclinical and clinical studies for multiplex measurement of diagnostic, prognostic and treatment-related biomarkers.

**Methods:**

We have established an SRM assay for multiplex analysis of 7 enzymes of the glycolysis pathway which is already known to be affected in human schizophrenia and in the widely-used acute PCP rat model of schizophrenia. The selected enzymes were hexokinase 1 (Hk1), aldolase C (Aldoc), triosephosphate isomerase (Tpi1), glyceraldehyde-3-phosphate dehydrogenase (Gapdh), phosphoglycerate mutase 1 (Pgam1), phosphoglycerate kinase 1 (Pgk1) and enolase 2 (Eno2). The levels of these enzymes were analyzed using SRM in frontal cortex from brain tissue of PCP treated rats.

**Results:**

Univariate analyses showed statistically significant altered levels of Tpi1 and alteration of Hk1, Aldoc, Pgam1 and Gapdh with borderline significance in PCP rats compared to controls. Most interestingly, multivariate analysis which considered the levels of all 7 enzymes simultaneously resulted in generation of a bi-dimensional chart that can distinguish the PCP rats from the controls.

**Conclusions:**

This study not only supports PCP treated rats as a useful preclinical model of schizophrenia, but it also establishes that SRM mass spectrometry could be used in the development of multiplex classification tools for complex psychiatric disorders such as schizophrenia.

## Background

Schizophrenia is a psychiatric disorder of uncertain etiology that affects about 1% of the world's population. It is likely to be caused by a complex crosstalk between genetic, neurodevelopmental and environmental factors [1] resulting in the differential expression of several genes and proteins. The multi-factorial characteristic of schizophrenia not only hinders its biochemical understanding and characterization, but makes it difficult to establish preclinical models, which are needed urgently for testing of existing and novel therapies [2]. The current evaluation of preclinical models for psychiatric disorders is mostly based on readouts following behavioral tests which simulate particular features or symptoms of the disease. However, characteristic schizophrenia symptoms, such as hallucinations, delusions and disorganized thoughts cannot be assessed using these tests. In contrast, characterization of these models using molecular fingerprinting approaches can be translated directly between preclinical and clinical studies.

The blockade of NMDA receptors using drugs such as phencyclidine (PCP) and ketamine is widely used to mimic schizophrenia in rodents. The psychosis-inducing effects of these drugs in humans and the widespread nature of the NMDA receptor in the brain result in multiple effects which may resemble the multifactorial features of schizophrenia [3]. Previous studies have shown that PCP-treated rats present altered glucose utilization in the limbic regions of their brains [4,5], consistent with findings from *post mortem *brain studies of schizophrenia patients [6].

**Figure 1:**
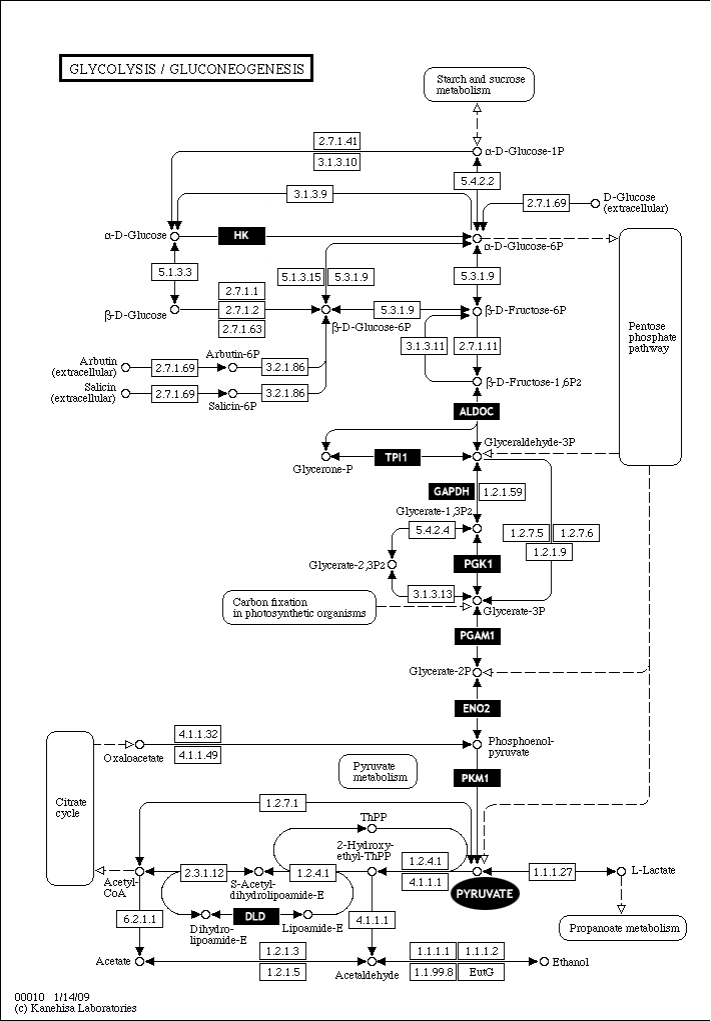
**Glycolysis metabolic pathway**. The differentially expressed enzymes and metabolites revealed by proteomics in post-mortem human brains from schizophrenia patients are contrasted in black (by KEGG - http://www.genome.jp/kegg).

Several brain regions collected from schizophrenia patients *post mortem *have been analyzed by other large-scale profiling techniques such as transcriptomic microarrays [7,8], and proteomic methods including two-dimensional gel electrophoresis and shotgun mass spectrometry [9,10]. These analyses have shown consistent effects on glycolysis enzymes [11] such as hexokinase 1 (Hk1), aldolase C (Aldoc), triosephosphate isomerase (Tpi1), glyceraldehyde-3-phosphate dehydrogenase (Gapdh), phosphoglycerate mutase 1 (Pgam1), phosphoglycerate kinase 1 (Pgk1) and enolase 2 (Eno2) (Figure 1[12]). These data also support previous findings of impaired glucose handling in schizophrenia brains [13-15]. Although impairments in energy metabolism seem to be a common trait of psychiatric disorders [16], disturbances in glycolysis appear to be more specific to schizophrenia [11]. On the other hand, effects on oxidative phosphorylation have been reported more frequently in brains from patients with major depressive disorder [17]. It is a requirement that preclinical models present similar traits to the disease in question. Considering the largely discovered dysregulation of glycolysis in schizophrenia brains as well as the evidence of differential glucose handling in preclinical models, the targeted analysis of glycolysis enzymes is one of the necessary steps in the molecular characterization of preclinical models of schizophrenia.

Most targeted protein expression analyses are performed using either Western blotting (WB) or enzyme-linked immunosorbent assay (ELISA) approaches. Although these methods are effective, both are dependent on antibody availability and neither are suitable for large-scale validation in their standard formats. Moreover, depending on the quality of the antibody employed, WB analysis is only considered to be a semi-quantitative method [18]. An alternative for targeted protein expression analysis is selective reaction monitoring (SRM) mass spectrometry [or multiple reaction monitoring (MRM)]. SRM is a mass spectrometry-based technique which can accurately measure the relative or absolute concentrations of selected peptides/proteins down to the attomolar range. In addition, SRM can be multiplexed, allowing the measurement of peptides from several proteins in a single experiment. Therefore, this method has the capability of measuring all components in a metabolic pathway in a single sample.

In this study, we have performed SRM mass spectrometry analysis of the seven glycolysis enzymes listed above in brain tissues of PCP-treated (1.0 and 2.5 mg/kg) and control rats. The resulting protein levels were then assessed using univariate and multivariate analyses, which showed that the acute PCP model reproduces some of the glycolytic deficits seen in schizophrenia at the molecular level.

## Results and discussion

**Figure 2:**
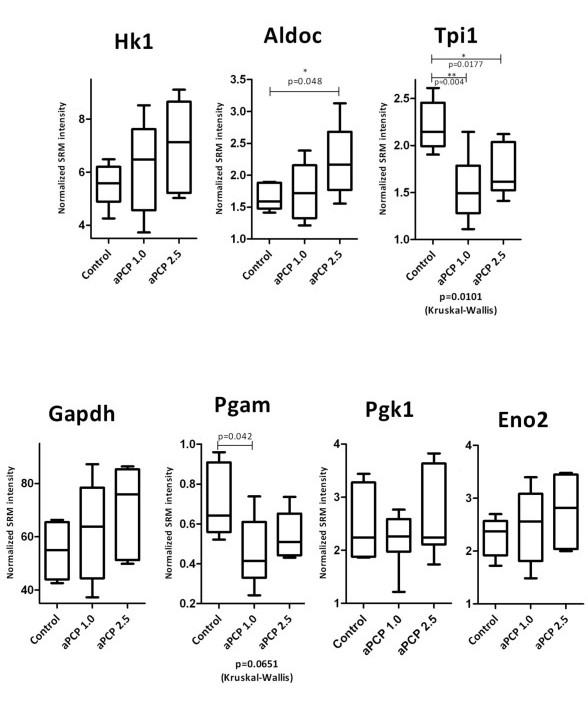
**Univariate analysis of glycolysis enzymes**. Mass spectrometry intensities are normalized relative to the spiked enolase peptides.

SRM data for the seven analyzed glycolytic enzymes in rats treated acutely with PCP showed significant differences in the levels of Tpi1 among the 3 groups (p = 0.0101 by the Kruskal-Wallis test) and a trend toward change for the model for Pgam1 (p = 0.065). The source of the change was identified by comparing the two versions of the model to the control rats with corrected Mann Whitney U p-values (Figure 2). The levels of Tpi1 and Pgam1 were decreased in the frontal cortex of PCP rats consistent with findings from human *post mortem *schizophrenia studies [19]. The levels of Aldoc, Hk1, Gapdh, Eno2 and Pgk1 were not found to be significantly altered in contrast with previous studies of schizophrenia in humans which found significant changes in the levels of these proteins [19-21].

As a multifactorial disorder, schizophrenia is likely to be a consequence of several molecular differences of small effect [22] triggered by environmental factors [23]. These subtle and sometimes even non-significant molecular differences may be one of the reasons why it has been difficult to replicate and even determine particular genes or biomarkers responsible for schizophrenia [24]. However, the combination of these small molecular differences may lead to major biological disturbances, especially if the differentially expressed molecules belong to the same biochemical pathway. We were therefore interested in determining whether the combination of the differential expression of glycolytic enzymes can be used to differentiate between controls and PCP treated groups despite the lack of statistical significance at the univariate level. In addition, we aim to check whether SRM is a suitable proteomic tool for this task.

**Figure 3:**
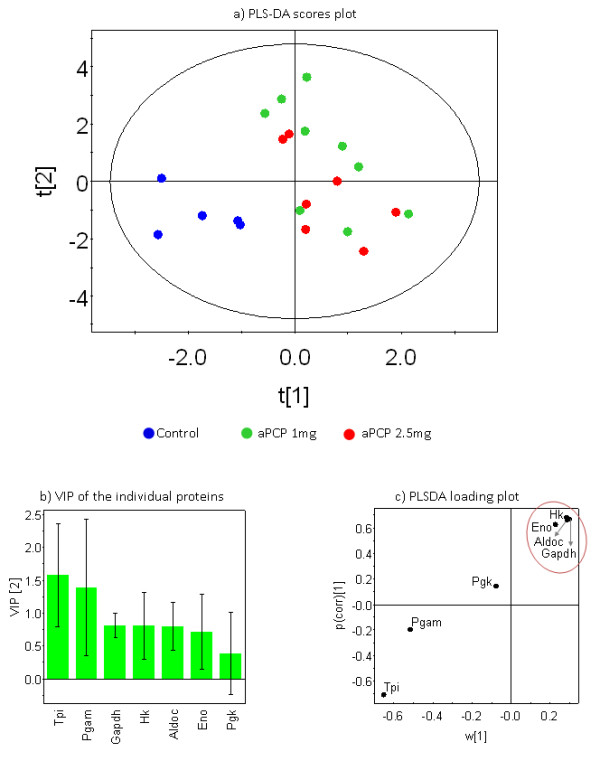
**Multivariate analyses of glycolysis enzymes analyzed by SRM**. a) Partial least squares discriminant analysis (PLS-DA) scores plot shows the distribution of the different samples in two dimensions, it should be noted that both PCP groups were treated as one class. Good separation was achieved between the model and the control. Also, the plot shows little difference between the samples in the 1 mg and 2.5 mg of PCP models. b) Variable influence on projection (VIP) bar chart shows that Tpi is most important to the separation, as shown in the scores plot. c) The loading plot also identifies Tpi as an important analyte for separation and shows that it is reproducible across samples in a group. Gapdh, Hk, Aldoc and Eno (inside red circle) cluster tightly together.

In the scores plot (Figure 3a), partial least squares discriminant analysis (PLS-DA) projects all the proteomic intensity data into a multi-dimensional space according to the number of analyzed variables - seven in this case. The plot as a whole is rotated in order to maximize the separation between the analyzed groups. This is then compressed down and presented on a two dimensional plot. Based on the data of seven glycolytic enzymes, this data provides a clear separation between the PCP model and the control group as seen on the scores plot (Figure 3a). The mathematical model suggests that an injection of 1 mg of PCP is similar to 2.5 mg of PCP as the model highlights no separation between the different PCP injections (Figure 3a). This suggests that the same molecules were affected at both dosages, most likely because the same molecular pathways were triggered above a threshold dosage of PCP. Figure 3b shows the influence of the proteins on the projection. The values explain the contribution of each protein to the separation in the scores plot (Figure 3a). Tpi and Pgam were the highest contributors to this separation, although Tpi was more robust than Pgam, which is consistent with the univariate findings that showed Tpi to be significantly different between animal model and control, with Pgam trending toward significance. The PLS-DA loading plot (Figure 3c) presents the relationship between the different proteins and how they relate to the separation on the scores plot (Figure 3b), it showed that Gapdh, Hk, Aldoc and Eno were clustered together. This suggests a strong correlation of their intensities and may indicate a link in the abundance of these proteins in the tissue analyzed (Figure 3c). This is interesting as none of these molecules showed statistically significant differences at the univariate level. However, the multivariate model suggests that they interact as components of the same pathway in distinguishing the PCP and control groups.

The translation of academic findings to the clinic is now a major objective of biomarker validation studies, especially in support of drug discovery. The Food and Drug Administration (FDA) has now called for efforts to modernize methods, tools and techniques for the purpose of delivering more efficacious and safer drugs [25,26]. SRM mass spectrometry can be optimized for performing high throughput multiplex analyses, which is robust and user friendly for use in the clinical environment, as stated above. Moreover, we have used the multiplex SRM mass spectrometry approach here to determine molecular phenotypes of a pre-clinical model of schizophrenia. This demonstrated the potential of translating this model for use in drug discovery with a set of companion biomarkers representing the glycolytic pathway to facilitate this process.

The validation of large scale proteomic profiling results has been a bottle neck in biomarker discovery. This is due in part to the lack of a robust technological pipeline connecting discovery with validation and translation, which is applicable to a clinical setting [27]. Here, we have shown that SRM mass spectrometry is a useful platform for achieving this goal. This approach has already been used in clinical studies such as those screening for the levels of dihydroartemisinin (DHA) in plasma of malaria patients [28], for determining the levels of apolipoproteins in human plasma [29] and for cancer biomarker screening [30]. Finally, although in a small number of samples, the results of the analysis support a role for the PCP rat as a good model for some aspects of schizophrenia, including metabolic dysfunction, as this has shown dysregulation of the glycolysis-related enzymes as previously shown in brain tissue from schizophrenia patients.

Limitations of our study include the number of analyzed samples. Replication for validating the biological interpretation here is suggested, which can also confirm the validity of our assessment using PLS-DA. Methodologically, Tpi1 was only analyzed by one peptide. All other possible Tpi1 peptides were neither proteotypic [31] nor quantotypic [32] as well as not prone to post-translational modifications as verified by PHOSIDA http://www.phosida.com[33].

## Conclusions

Previous studies have shown alterations in glucose uptake in the brain following acute PCP treatment, with an initial increase within 3 hours, followed by a decrease lasting up to 2 days [4]. This finding was recently corroborated by in vivo NMR spectroscopy [5], which showed the increased glucose uptake peaked 20 minutes after PCP injection. However it is not known whether the increase in glucose concentration is due to increased uptake through the blood-brain barrier, to decreased utilization of glucose by the brain, or both. Nevertheless, these observations support our results considering the overall differential expression of glycolytic enzymes. The multivariate model demonstrated how non-significant variations of several components of a pathway can lead to a significant biological response, as observed previously on the behavior of PCP treated animals [34,35]. Such effects may also be likely in the case of schizophrenia as conflicting reports have been published on the expression changes of individual molecules (Table 1 in [9]).

**Table 1:** Glycolytic enzymes candidates and peptides analyzed

Protein	Peptide sequence/charge
**Hk1**	EGLLFEGR.2
	GAALITAVGVR.2
	GAALITAVGVR.3
	ITPELLTR.2
	NILIDFTK.2
**Aldoc**	DDNGVPFVR.3
	DNAGAATEEFIK.2
**Tpi1**	HIFGESDELIGQK.3
**Gapdh**	LVINGKPITIFQER.3
	VIPELNGK.2
**Pgam1**	HYGGLTGLNK.3
	VLIAAHGNSLR.2
**Pgk1**	ITLPVDFVTADK.2
	LGDVYVNDAFGTAHR.3
**Eno2**	FTANVGIQIVGDDLTVTNPK.2
	IEEELGEEAR.2
	LGAEVYHTLK.2
	LGAEVYHTLK.3

Our findings warrant further biological research on the analysis of glycolytic enzymes in other schizophrenia models, including investigating the effects of drug treatments. Further technical developments should be directed towards the use of SRM as a multiplex tool for diagnostic purposes. In addition, pathways representing other aspects of schizophrenia could be developed using the SRM format, especially in cases of models where other pathways are known to be modified.

## Methods

### Samples

Male, adult Lister Hooded rats (Harlan, Horst, The Netherlands), weighing 180-220 g were housed in groups of three to four under standard laboratory conditions at a temperature of 21°C (± 1°C). Rats were maintained on a 12-h/12-h light/dark cycle (lights on at 08:00). Experiments were conducted during the light cycle. Food and water were available *ad libitum*. All experiments were performed in accordance with the European Communities Council Directives (86/609/EEC) and were approved by the policies of the ethical committee of Johnson and Johnson Pharma (Beerse, Belgium). Rats received an acute subcutaneous injection of either 1 mg/kg (n = 9) or 2.5 mg/kg (n = 7) PCP as these dosages have been shown previously to significantly enhance locomotion in open field testing [36]. PCP (Johnson and Johnson Pharma, Beerse, Belgium) was dissolved in 0.9% saline. The control group (n = 5) received an equivalent volume of saline (Baxter, Belgium). Sixty minutes after PCP or saline administration, rats were decapitated and brains were dissected immediately on ice to obtain frontal cortex (FC) samples, which were stored at -80°C until further use.

### Protein extraction

Brain tissue samples were sonicated for 2 minutes in a buffer containing 7 M urea, 2 M thiourea, 4% CHAPS, 2% ASB14, 70 mM dithiothreitol (DTT) (all from Sigma) at a 5:1 (v:w) ratio [37] for proteome extraction. Samples were vortexed for 30 minutes and centrifuged for 3 minutes at 17000 g at 4°C. Protein concentrations in the supernatants were determined using a Bradford assay (Biorad, UK). Extracted proteins (100 mg) were precipitated overnight using cold acetone (-20°C) and centrifuged for 10 min at 17000 g at 4°C. The supernatants were discarded and the pellets dissolved in 50 mM ammonium bicarbonate. Next, sulfhydryl groups were reduced with 5 mM DTT at 60°C for 30 minutes followed by alkylation with 10 mM iodacetamide in the dark at 37°C for 30 minutes. Each resultant proteome was digested using trypsin at a 1:50 (w:w) ratio for 17 hours at 37°C. Reactions were stopped by addition of 8.8 M HCl at a 1:60 (w:w) ratio. The concentrations of the digested proteins (also referred to as the peptidome) were adjusted to 0.1 μg/μL by addition of the appropriate amounts of 0.1% formic acid. Samples were spiked with 25 fmol of yeast enolase for data normalization.

### Mass spectrometry experiments

Peptidomes were injected in duplicate into a nanoUltra performance liquid chromatography instrument containing a BEH-130 C18 column (75 um × 200 mm) at a flow rate of 0.3 μL/min, coupled online to a Xevo-triple-quadrupole mass spectrometer (Waters; Milford, MA, USA). The separation buffers were A) 0.1% formic acid and B) acetonitrile in 0.1% formic acid. For separation of peptides, the following 48 min gradient was applied: 97/3% (A/B) to 60/40%B in 30 min; 60/40% to 15/85% in 2 min; 5 min at 15/85%; returning to the initial condition in 1 min. Selected peptides (Table 1) eluting from the nanoLC were measured in SRM mode using an electrospray voltage of 22 kV and a cone voltage of 30 V. All SRM functions had a 2 min window of the predicted retention time and scan times were 20 milliseconds. At least 2 SRM transitions per peptide were measured. The collision energy for each transition was optimized using Skyline software (MacCoss Lab Software; Seattle, WA, USA) [38] based on the equation: CE = 0.034*m/z +3.314. Acquired data were processed and quantified using TargetLynx (Waters). Mass spectral intensities of peptides from the target proteins were averaged and normalized against yeast enolase intensities.

### Statistical analyses

To account for potential non-Gaussian distributions, data were assessed using the Kruskal-Wallis test to find if any differences were present among the three groups. Significant differences between the two dosages of PCP and control groups were calculated using corrected Mann Whitney U p-values, applying the Benjamini-Hochberg method for multiple testing. All tests were two-tailed and conducted in Prism 5 (Graphpad Software Inc; La Jolla, CA, USA) or R 2.13 (The R Foundation for Statistical Computing, Vienna, Austria).

For the multivariate analyses, partial least squares discriminant analysis (PLS-DA) was used (Simca-P + 12; Umetrics; Umea, Sweden). PLS-DA is a supervised method for fine tuning a principle component analysis (PCA) model to maximize separation between two groups of observations. The protein intensities were uploaded to the software which assembled these linearly to form PLS components that were plotted in multidimensional space. These plots were then reduced to the principle components which are presented as a two-dimensional PLS-DA scores plot [39]. In addition to this 2D plot, the software calculates the variable influence on projection (VIP) which assesses the contribution of each variable on the separation observed on the PLS-DA scores plot [40]. Lastly, the loading plot (S-plot) is presented. The x-axis represents the contribution of each variable to the separation). Variables positioned furthest from the centre have the strongest contribution. The data plotted along the y-axis show the correlation of each variable from sample to sample (the further the variable is from the centre, the stronger the correlation [41]).

## Competing interests

HR, PCG and BA are consultants for Psynova Neurotech Ltd. This work has been supported by Stanley Medical Research Institute.

## Authors' contributions

The study was conceived by DMS who executed the LC-SRM experiments and analyses. DMS wrote the manuscript helped by PCG. MA and LWH performed statistical analyses and contributed to the manuscript writing. AE and NA conducted animal experiments and AE prepared protein and peptide samples. NA, IL and PP provided the tissue samples. BA developed computation algorithms for SRM data analyses. HR, SB and PCG contributed to the manuscript writing and scientific discussions. All authors read and approved the final manuscript.
